# The gut resistome as a potential determinant of immunotherapy response: antibiotics, immunometabolism, and precision oncology

**DOI:** 10.3389/fmicb.2026.1835588

**Published:** 2026-07-08

**Authors:** Min-Kyung Joo, Jihoon Tak, Suhyeon Ha, Kyung-sik Yoon, Jung-Man Namgoong, Hye Ok Kim

**Affiliations:** 1 Experimental Immunology Branch, Center for Cancer Research, National Cancer Institute, NIH, Bethesda, MD, United States; 2 Department of Anesthesiology, Critical Care and Pain Medicine, McGovern Medical School, The University of Texas Health Science Center at Houston, Houston, TX, United States; 3 Department of Pediatric Surgery, Asan Medical Center Children’s Hospital, University of Ulsan College of Medicine, Seoul, Republic of Korea; 4 Department of Biochemistry and Molecular Biology, College of Medicine, Kyung Hee University, Seoul, Republic of Korea; 5 Department of Medicine, College of Medicine, Kyung Hee University, Seoul, Republic of Korea; 6 Clinical Research Institute, Kyung Hee University Medical Center, Seoul, Republic of Korea

**Keywords:** antibiotics, gut microbiome, immune checkpoint inhibitors, immunotherapy, resistome

## Abstract

Immune checkpoint inhibitors (ICIs) have transformed cancer therapy, yet their efficacy can be compromised by systemic antibiotic exposure and the resulting disruption of the gut microbiome. Across several tumor types, antibiotic use near the initiation of ICI therapy has frequently been associated with reduced progression-free and overall survival. Emerging data suggest that, beyond taxonomic shifts, antibiotic exposure is often accompanied by expansion of the gut resistome, the collective pool of antibiotic resistance genes. Antibiotic-associated dysbiosis and resistome enrichment are linked to alterations in short-chain fatty acid production, bile acid signaling, and microbial purine metabolism, pathways known to shape antigen presentation, T-cell differentiation, and immune tone. Accordingly, gut resistome profiling should be considered an emerging candidate biomarker. Potential strategies to restore a favorable gut ecosystem include dietary modulation, microbiome-based therapies such as probiotics or fecal microbiota transplantation, and emerging anti-resistance approaches designed to limit resistome expansion. Together, these findings support a resistome-centered framework for patient stratification and microbiome-targeted interventions in precision immune-oncology.

## 1 Introduction

Immune checkpoint inhibitors (ICIs) have transformed cancer therapy and revealed that treatment response is shaped not only by tumor-intrinsic factors but also by the host environment (Gopalakrishnan et al., 2018; Ribas and Wolchok, 2018). Clinical studies across multiple tumor types have frequently reported that systemic antibiotic exposure near immunotherapy initiation is associated with poorer survival outcomes, although the strength of this association is heterogeneous across study designs, cancer types, and analytic models (Huang et al., 2019, 2021; Petrelli et al., 2020; Tsikala-Vafea et al., 2021). Most studies interpret this effect in terms of changes in microbial composition. Loss of diversity or depletion of specific bacterial taxa has been suggested to explain impaired immunotherapy responses (Gopalakrishnan et al., 2018; Pietrzak et al., 2022). However, this descriptive approach does not fully account for the variability observed across tumor types, antibiotic classes, and exposure windows. Similar antibiotic regimens can produce markedly different effects across cancer types and antibiotic classes (Qiu et al., 2022). These inconsistencies suggest that microbial function, rather than composition alone, plays a critical role.

In this context, the gut resistome provides a complementary framework. The gut resistome refers to the full collection of antibiotic resistance genes (ARGs) present within the intestinal microbiota (Kim and Cha, 2021; van Schaik, 2015). Importantly, the resistome reflects the ecological history of antibiotic exposure and captures the functional capacity of the microbial community (Radovanovic et al., 2023). Unlike species abundance, which can partially recover after treatment, resistance gene profiles often persist for extended periods (Kang et al., 2021; Tigabu and Leung, 2026; Zhou et al., 2025). As a result, the resistome may represent a durable record of microbial selection and a functional marker of long-lasting ecological and metabolic remodeling after antibiotic exposure (Dongre et al., 2025; Singh et al., 2019).

Expansion of antibiotic resistance genes reshapes microbial metabolic pathways that regulate immune function (Clavijo-Salomon and Trinchieri, 2025; Perl et al., 2025; Wright, 2007). These changes influence short-chain fatty acid production, bile acid metabolism, and purine signaling (Liu et al., 2025; Perl et al., 2025). Each of these pathways contributes to dendritic cell activation, antigen presentation, and T-cell programming (Buck et al., 2017; Pearce and Pearce, 2013; Xie et al., 2025). When these metabolic circuits are disrupted, immune priming becomes inefficient, and T-cell exhaustion programs are more likely to emerge during checkpoint blockade (Lei et al., 2025). Human studies suggest that ARG patterns may stratify immunotherapy outcomes more consistently than species-level abundance (Gopalakrishnan et al., 2018; Iwan et al., 2024), although direct resistome-centered evidence remains limited, and prospective validation remains needed.

This review examines clinical evidence linking antibiotic exposure to immunotherapy outcomes and reinterprets these findings through a resistome-centered conceptual framework. We discuss how timing and antibiotic class influence ICI efficacy. We then describe how resistome-driven changes in short-chain fatty acids (SCFAs), bile-acid metabolism, and purine metabolism influence antigen presentation and T-cell programming (Liu et al., 2025; Perl et al., 2025). Finally, we review dietary, microbial, and anti-resistance strategies that may stabilize resistome states and help preserve immunotherapy effectiveness. It is part of the microbiome-based therapy that clinicians can actively modify through antibiotic stewardship, dietary interventions, fecal microbiota transplant, defined microbial consortia, and new anti-resistance approaches (Clavijo-Salomon and Trinchieri, 2025; Ting et al., 2022). The overall goal is to shift microbiome-immunotherapy research from descriptive associations toward a precision-medicine framework in which the gut resistome may serve as a measurable candidate biomarker and mechanistic readout of treatment-relevant microbiome disruption.

For clarity, this review distinguishes three levels of evidence. First, the association between antibiotic exposure near ICI initiation and poorer clinical outcomes is supported by cohort studies and meta-analyses. Second, experimental and translational studies support that antibiotics alter microbiome structure, ARG burden, SCFA availability, bile acid metabolism, purine signaling, and immune tone. Third, we present the resistome-centered model as a working hypothesis: ARG expansion may serve as a functional biomarker, and potentially a contributor, to impaired ICI response.

## 2 Part I. Clinical links between antibiotics, the gut resistome, and ICI outcomes

### 2.1 Clinical evidence linking antibiotic exposure to ICI outcomes across cancer types

Antibiotic exposure has emerged as a recurrent clinical correlate of ICI outcomes, although the magnitude and even statistical significance of this association vary across tumor types, treatment contexts, exposure definitions, and patient cohorts (Crespin et al., 2023; Tsikala-Vafea et al., 2021; Wilson et al., 2020). To clarify these patterns, a growing body of studies has systematically evaluated how antibiotic use before or during ICI therapy influences response rates, progression, and survival. Section 1 organizes clinical evidence across major tumor types, highlighting both the shared trends and the tumor-specific nuances that define the relationship between antibiotics and ICI efficacy. Table 1 summarizes the major clinical studies by cancer type, exposure window, and associated ICI outcomes.

**TABLE 1:** Clinical evidence linking antibiotic exposure to immune checkpoint inhibitor outcomes across cancer types.

Study	Cancer type	ICI class / agent	ATB window	Clinical outcomes (no abx vs. abx)	
Hakozaki et al., 2019	NSCLC	PD-1	Pre-ICI	↓PFS (4.4 vs. 1.2, *P* = 0.04) ↓OS (NR vs. 8.8, *P* = 0.037)	PFS HR = 2.02 (*P* = 0.19)
Derosa et al., 2018	NSCLC	PD-1	−30 days	↓PFS (3.8 vs. 1.9, *P* < 0.05) ↓OS (24.6 vs. 7.9, *P* < 0.05)	PFS HR = 1.5 (*P* = 0.03) OS HR = 4.4 (*P* < 0.01)
Zhao et al., 2019	NSCLC	PD-1	−60 days	↓PFS (9.63 vs. 3.73, *P* = 0.0006) ↓OS (21.87 vs. 6.00, *P* = 0.015)	PFS HR = 0.32 (*P* < 0.0001) OS HR = 0.35 (*P* = 0.009)
Ruiz-Patiño et al., 2020	NSCLC	PD-1/PD-L1	−30 days	↓PFS (2.7 vs. 1.9, N.S.) ↓OS (40.6 vs. 20.3, *P* < 0.05)	–
Nyein et al., 2022	NSCLC	PD-1/PD-L1	−30 or −60 days	↓OS (16.3 vs. 12.4, *P* = 0.145)	OS HR = 1.35 (*P* = 0.140)
Martinez-Mugica Barbosa et al., 2022	NSCLC	PD-1/PD-L1	−60 days	↓PFS (5.5 vs. 4, *P* = 0.38) ↓OS (15.4 vs. 12.9, *P* = 0.84)	–
Deng et al., 2024	NSCLC	Anti–PD-1	−60 days	↓PFS (10.4 vs. 11.0, N.S.) ↓OS (29.0 vs. 22.5, N.S.)	PFS HR = 0.945 (*P* = 0.663) OS HR = 1.396 (*P* = 0.143)
Elkrief et al., 2024	NSCLC	PD-1/PD-L1	−60 days	↓PFS (2.8 vs. 1.9, *P* = 0.058) ↓OS (13 vs. 5.3, *P* = 0.002)	PFS HR = 1.4 (*P* = 0.058) OS HR = 1.47 (*P* = 0.002)
Ochi et al., 2021	NSCLC	PD-1/PD-L1	Mixed	↓PFS (3.5 vs. 3.5, *P* = 0.287) ↓OS (16.1 vs. 11.7, *P* = 0.028)	–
Wilson et al., 2020	NSCLC	PD-1/PD-L1	−60 days	–	PFS HR = 1.64 (*P* < 0.05) OS HR = 2.00 (*P* < 0.05)
Elkrief et al., 2019	Melanoma	PD-1	−30 days	↓OS (18.3 vs. 7.5, *P* = 0.04)	OS HR = 0.27 (*P* = 0.0)
Chorti et al., 2024	Melanoma	PD-1	−60 days	↓PFS (7.8 vs. 3.0, *P* < 0.05) ↓OS (51.5 vs. 17.3, *P* < 0.05)	PFS HR = 1.75 OS HR = 1.64
Hirner et al., 2025	Melanoma	PD-1	−7 days	↑OS (*P* < 0.05)	–
Poizeau et al., 2022	Melanoma	PD-1/PD-L1	−90 days	–	OS HR = 1.01
Mohiuddin et al., 2021	Melanoma	PD-1/PD-1	−90 days	–	OS HR = 1.95 (*P* < 0.001)
Lalani et al., 2020	RCC	PD-1/PD-L1	−60 days	–	PFS HR = 1.96 (*P* = 0.007) OS HR = 1.44 (*P* = 0.27)
Derosa et al., 2018	RCC	PD-1/PD-L1	−30 days	↓PFS (7.4 vs. 1.9, *P* < 0.05) ↓OS (30.6 vs. 17.3, *P* < 0.05)	PFS HR = 3.1 (*P* < 0.01) OS HR = 3.5 (*P* = 0.03)
Luo et al., 2022	RCC	PD-1	−60 days	–	PFS HR = 2.10 OS HR = 1.69
Ishiyama et al., 2021	Urothelial	Pembrolizumab	Pre-ICI	↓PFS (8.9 vs. 1.1, *P* < 0.001) ↓OS (19.5 vs. 2.3, *P* < 0.001)	–
Febriyanto et al., 2024	Urothelial	PD-1	−60 days	–	PFS HR = 1.40 OS HR = 1.45
Hopkins et al., 2020	Urothelial	Atezolizumab	−30 days	–	PFS HR = 1.24 OS HR = 1.44
Serpas Higbie et al., 2022	CRC	PD-1	Any time point from −90 to +180 day	↓PFS (*P* = 0.36) ↓OS (*P* = 0.29)	–
Alotaibi et al., 2024	GI cancers	PD-1	Mixed	–	PFS HR = 1.81 (*P* < 0.0007) OS HR = 1.92 (*P* = 0.004)
Petrelli et al., 2020	Pan-cancer	PD-1/PD-L1	−60 to +60 days	–	PFS HR = 1.53 (*P* < 0.01) OS HR = 2.07 (*P* < 0.01)
Tsikala-Vafea et al., 2021	Pan-cancer	PD-1/PD-L1	−60 to +60 days	–	PFS HR = 1.36 (NSCLC) OS HR = 1.94 (NSCLC) PFS HR = 6.52 (Melanoma) OS HR = 4.42 (Melanoma) PFS HR = 2.42 (RCC) OS HR = 3.50 (RCC)
Huang et al., 2019	Pan-cancer	PD-1/PD-L1	Pre-ICI	–	PFS HR = 1.84 (*P* < 0.001) OS HR = 2.37 (*P* < 0.001)
Pinato et al., 2019	Pan-cancer	PD-1/PD-L1	Pre-ICI	↓OS (26 vs. 2, *P* < 0.001)	

NSCLC, non-small cell lung cancer; ICI: immune checkpoint inhibitors; PFS, progression-free survival time; OS, overall survival time; N.S., no significant differences; NR, not-reached.

#### 2.1.1 Non-small cell lung cancer (NSCLC)

Clinical studies over the past decade have shown that systemic antibiotic exposure is associated with reduced efficacy of immune checkpoint inhibitors across several major cancer types (Huang et al., 2019, 2021; Petrelli et al., 2020; Pinato et al., 2019). Multicenter cohort studies and meta-analyses generally support an adverse association between antibiotic exposure and ICI outcomes in non-small cell lung cancer (NSCLC; Hakozaki et al., 2019; Martinez-Mugica Barbosa et al., 2022; Nyein et al., 2022; Zhao et al., 2019). Elkrief et al. (2024) reported that antibiotics given within 60 days before immunotherapy shortened PFS (progression-free survival time) from 2.8 to 1.9 months and OS (overall survival time) from 13 to 5.3 months. These findings are supported by several meta-analyses in which antibiotic-exposed NSCLC patients consistently showed inferior outcomes, with OS hazard ratios (HRs) ranging from approximately 1.9 to 2.1 and PFS HRs between about 1.5 and 1.8 (Huang et al., 2019, 2021). Across NSCLC cohorts, reduced OS and PFS are most consistently observed following exposure to β-lactam antibiotics, including penicillin and piperacillin/tazobactam, and to fluoroquinolones (Deng et al., 2024; Qiu et al., 2022).

#### 2.1.2 Melanoma

In melanoma, the interval between antibiotic exposure and immunotherapy initiation appears to influence outcomes. Several melanoma studies report shorter OS and/or PFS among antibiotic-exposed patients (Chorti et al., 2024; Huang et al., 2019; Mohiuddin et al., 2021), whereas other analyses have shown null or time-window-dependent associations (Deng et al., 2024; Serpas Higbie et al., 2022), suggesting that the evidence is clinically suggestive but not fully uniform. Patients who began immunotherapy at least 1 week after completing antibiotics had better survival than those who started treatment sooner, suggesting that early immune priming is highly sensitive to microbiome disruption (Hirner et al., 2025). Reduced response rates are also observed in patients exposed to macrolides or β-lactams, suggesting that the antibiotic class contributes to the magnitude of ICI impairment (Elkrief et al., 2019). In addition, prior antibiotic use has been associated with a higher incidence of *Clostridioides difficile* colitis during ICI therapy, further reflecting treatment-mediated colitis following microbiome perturbation (Mohiuddin et al., 2021). These findings suggest that the biological effect of antibiotics may be most disruptive immediately before immunotherapy, when antigen-presenting cell activation and CD8 T-cell priming are critical for effective ICI initiation.

#### 2.1.3 Renal cell carcinoma (RCC)

Renal cell carcinoma exhibits a comparable pattern, but with an important nuance. In a key study, antibiotics had no measurable effect in patients receiving non-immunotherapy regimens, indicating that antibiotics interact specifically with immune-mediated mechanisms (Lalani et al., 2020). Meta-analysis data support that antibiotic exposure before ICI worsens OS and PFS outcomes in patients with renal cell carcinoma (Luo et al., 2022; Tsikala-Vafea et al., 2021). RCC cohorts specifically highlight that penicillin- or cephalosporin-based regimens produce the strongest decline in ICI-treated survival, a pattern consistent with β-lactam–driven ecological disruption, and these studies report beta-lactam/β-lactamase inhibitor combinations (39.3%), fluoroquinolones (25%), macrolides (10.7%), and tetracyclines (10.7%) as the most common antibiotic classes used (Lalani et al., 2020).

#### 2.1.4 Urothelial carcinoma

In urothelial carcinoma, although the dataset remains limited, retrospective, and vulnerable to residual confounding, most available studies suggest a detrimental association. A dedicated meta-analysis of immunotherapy-treated metastatic urothelial carcinoma reported increased OS and PFS HRs in patients treated with antibiotics (Febriyanto et al., 2024). An additional cohort of pembrolizumab-treated patients also demonstrated shorter survival among those receiving antibiotics, even after adjusting for performance status and metastatic burden (Ishiyama et al., 2021). Hopkins et al. (2020) reported that among patients with urothelial carcinoma treated with atezolizumab, 37% received penicillins, 37% received quinolones, and 36% received cephalosporins. The study found that antibiotic exposures, which disrupt the gut microbiota, were significantly associated with poorer immunotherapy outcomes. β-lactam–fluoroquinolone combinations have stronger negative effects on ICI outcomes compared with fluoroquinolone monotherapy (Febriyanto et al., 2024).

#### 2.1.5 Colorectal cancer (CRC): an important exception

Colorectal cancer has far more limited evidence regarding the impact of antibiotics on immunotherapy outcomes. The only colorectal cancer–specific study to date, conducted in MSI-H/dMMR metastatic disease, found no significant differences in response rate, PFS, or OS between antibiotic-exposed and unexposed patients (Serpas Higbie et al., 2022). A recent meta-analysis of gastrointestinal cancers, including CRC, reported that antibiotic exposure was associated with worse outcomes in GI tumors overall but did not provide CRC-specific analyses (Alotaibi et al., 2024). CRC has extensive evidence linking antibiotics and gut microbiota in carcinogenesis, but there are still no studies testing antibiotic class, exposure window, or resistome alterations specifically in ICI-treated CRC patients (Simin et al., 2020). This gap highlights the need for CRC-specific studies that measure functional resistome states rather than relying on species profiles alone.

#### 2.1.6 Interpreting the clinical evidence: heterogeneity and residual confounding

Overall, the clinical literature is directionally suggestive but not uniformly concordant. Although many retrospective studies and meta-analyses report worse survival among antibiotic-exposed patients receiving ICIs, several cohorts have shown attenuated, mixed, or non-significant associations, particularly when exposure windows are broadened, treatment backbones differ, or multivariable adjustment is applied. Importantly, antibiotic exposure is often intertwined with infection severity, hospitalization, steroid use, poor performance status, higher tumor burden, and later lines of treatment, all of which may independently predict inferior ICI outcomes. Additional heterogeneity arises from inconsistent definitions of antibiotic timing relative to ICI initiation, incomplete reporting of duration, dose, route, and class, and limited separation of ICI monotherapy from chemoimmunotherapy. Accordingly, antibiotic exposure should be interpreted not only as a potential biological modifier of ICI efficacy but also, in some settings, as a surrogate marker of a clinically more vulnerable population.

### 2.2 Antibiotic timing is a critical variable for risk stratification in immunotherapy

While the negative association between antibiotics and immunotherapy outcomes is well recognized, the risk is not uniform. Emerging data indicate that the timing and class of antibiotic exposure critically shape its impact, revealing that antibiotic effects extend beyond simple microbiome depletion (Pinato et al., 2019). These variables expose deeper biological mechanisms, particularly functional disruptions within the gut microbial ecosystem, that influence early immune priming. Section 2 examines how timing windows, antibiotic classes, and functional microbiome alterations refine risk assessment and move the field beyond taxonomy-focused interpretations.

#### 2.2.1 High-risk timing windows across cancers

Clinical studies across melanoma, non-small cell lung cancer, renal cell carcinoma, urothelial carcinoma, and colorectal cancer consistently show that the survival impact of antibiotics is not uniform but is strongly shaped by the timing of exposure (Huang et al., 2019; Petrelli et al., 2020; Pinato et al., 2019). Multiple analyses identify the period from approximately 60 days before the first ICI dose to the early weeks after initiation as the most vulnerable interval. In NSCLC, for example, antibiotics administered within 30 days before anti-PD-1 therapy reduced OS (Derosa et al., 2018; Ochi et al., 2021; Ruiz-Patiño et al., 2020). Meta-analytic work also found that antibiotic exposure during the −60 to +60 days interval was consistently associated with worse OS HRs and PFS HRs across multiple tumor types (Huang et al., 2019, 2021; Tsikala-Vafea et al., 2021). Wilson et al. (2020) reported that defining antibiotic exposure as within 42 days before the start of ICI revealed a greater detrimental effect on overall survival. Comparable window-dependent effects have been observed in melanoma, RCC, and urothelial carcinoma, where exposures clustered in the 30–60 days pre-ICI period consistently predict poorer response and survival (Chorti et al., 2024; Elkrief et al., 2024; Febriyanto et al., 2024; Lalani et al., 2020). In melanoma, the first study that directly tested the timing between antibiotics and ICIs showed that the negative effect of antibiotics depends on how soon ICIs are started afterward. Patients who waited at least 7 days, and especially 14 days, after finishing antibiotics had better overall survival than those who began ICIs within the first week (Hirner et al., 2025). Exposure to antibiotics in the 3-month window before anti-PD-1 antibody initiation showed no association with shorter OS or PFS (Poizeau et al., 2022). These data indicate that the detrimental impact of antibiotics is not static but is modulated by the interval between antibiotic exposure and the start of immunotherapy.

#### 2.2.2 The role of antibiotic class

The clinical signal becomes sharper when the antibiotic class is considered. In NSCLC cohorts in which classes were analyzed separately, β-lactam antibiotics, particularly penicillin-based regimens, after exposure windows of 60 days before to 30 days after ICI initiation, produced the most reproducible survival disadvantage (Pinato et al., 2019; Qiu et al., 2022). Penicillin class exposure significantly worsened overall survival and progression-free survival, whereas cephalosporins, carbapenems, and fluoroquinolones used in isolation did not demonstrate the same effect size (Qiu et al., 2022). Combinations of β-lactams with fluoroquinolones produced the poorest survival profiles in the same cohort. Similarly, Macrolides and fluoroquinolones show negative associations in melanoma and urothelial carcinoma (Elkrief et al., 2019; Febriyanto et al., 2024), although fluoroquinolone monotherapy occasionally demonstrates attenuated effects compared with β-lactam–based regimens. Mohiuddin et al. (2021) reported that the type of antibiotic administered influences patients’ responses to ICIs. Penicillin was associated with the most pronounced negative impact, followed by cephalosporins and quinolones. In contrast, vancomycin did not show any significant effect on patient survival (Mohiuddin et al., 2021). Together, these findings show that not all antibiotics carry the same level of risk and that the extent of microbial disruption plays a central role in determining how strongly ICIs are affected.

#### 2.2.3 Taxonomy-based biomarkers cannot explain these patterns

Although timing and antibiotic class are strong clinical variables, they also reveal the limitations of a taxonomic biomarker framework. The same antibiotic class and window produce different outcome magnitudes across tumor types, and some fluoroquinolone exposures show non-significant effects even within NSCLC cohorts (Lu et al., 2021). These inconsistencies cannot be fully explained by changes in microbial species abundance. Instead, they reflect functional alterations that antibiotics impose on the gut microbial ecosystem (Kang et al., 2021). Antibiotic exposure promotes the expansion of bacterial populations carrying specific resistance genes, producing durable expansion of β-lactamase, macrolide resistance, tetracycline resistance, or aminoglycoside resistance elements (Kang et al., 2021; Luchen et al., 2023). These shifts alter metabolic pathways that regulate antitumor immunity, including short-chain fatty acid fermentation, bile acid modification, and purine metabolism (Perl et al., 2025). These metabolic disruptions offer a functional explanation for why identical antibiotic exposures produce heterogeneous clinical outcomes across different cancers, and why a taxonomy-only framework fails to fully capture the biological consequences of antibiotic treatment.

#### 2.2.4 Preliminary evidence from resistome-centered studies

Resistome-centered data in melanoma provide a clinically suggestive, functionally informative example. Enrichment of *Akkermansia muciniphila* and *Faecalibacterium prausnitzii* characterizes responders to anti-PD1 therapy, who also display microbiome-derived metabolic profiles favorable to antitumor immunity (Gopalakrishnan et al., 2018; Routy et al., 2018). In contrast, non-responders display higher loads of β-lactamase, macrolide resistance, and tetracycline resistance genes independent of tumor type or prior clinical variables (Gopalakrishnan et al., 2018). Early NSCLC datasets further suggest that specific resistance determinants such as lnuC, ermG, msrD, fosA, and aph(6)-Id correlate with poorer progression-free and overall survival independent of antibiotic class or timing (Iwan et al., 2024). These observations indicate that functional genetic potential, rather than species identity, more accurately captures how the microbiome influences the early immune programming required for immunotherapy success.

#### 2.2.5 Persistence of resistome perturbation

Antibiotic-driven disturbances in the gut resistome often persist far longer than changes in species composition. Longitudinal metagenomic studies show that even short antibiotic courses can rapidly expand specific resistance genes and that these ARGs may remain elevated for weeks to months after treatment ends, despite partial taxonomic recovery (Kang et al., 2021). Experimental work also demonstrates that different antibiotic classes generate distinct resistance signatures that can persist within the microbial community, reflecting selective expansion of ARG-enriched strains rather than simple loss of diversity (Luchen et al., 2023). Together, these findings align with clinical observations that the 60-day pre-treatment interval is the most vulnerable period for patients starting ICIs, because resistome perturbations endure well beyond the clinical administration of antibiotics.

#### 2.2.6 From taxon-centric to resistome-centric risk stratification

Taken together, current clinical associations and mechanistic studies support a convergent model in which antibiotics alter the gut ecosystem in ways that may weaken its capacity to support antitumor immunity. Antibiotics alter the gut ecosystem in ways that weaken its capacity to support antitumor immunity (Clavijo-Salomon and Trinchieri, 2025). In melanoma, responders to anti-PD1 therapy consistently show lower resistance gene burden and metabolic signatures associated with enhanced antitumor immunity, even after accounting for taxonomic differences (Gopalakrishnan et al., 2018). *Bifidobacterium*, *Enterococcus*, *Faecalibacterium*, *Ruminococcus*, and *Clostridiales* have been reported to promote the infiltration of CD8 T-cells in the tumor microenvironment (Matson et al., 2018; Sivan et al., 2015; Vétizou et al., 2015). These findings suggest that the biological effect of antibiotics may be most disruptive immediately before immunotherapy, when antigen-presenting cell activation and CD8 T-cell priming are critical for effective ICI initiation.

In NSCLC, a high abundance of specific resistance genes, including lnuC, msrD, ermG, aph(6), and fosA, has been associated with shorter progression-free and/or overall survival in an exploratory cohort (Iwan et al., 2024). These observations underscore that resistance-gene content captures immunologically relevant functional shifts that taxonomic biomarkers miss. Because most clinical studies still treat antibiotics as a binary variable and rarely measure ARG load, existing models cannot fully explain why similar antibiotic exposures produce different outcome magnitudes across cancer types. A resistome-centric framework may offer a useful complementary link between antibiotic history, microbial function, and immune programming that shapes ICI efficacy.

### 2.3 Methodological framework for gut resistome profiling and clinical feasibility

To advance the gut resistome from a conceptual framework toward an investigational biomarker, standardized methodologies for ARG detection, quantification, and interpretation will be essential. Currently, two primary approaches dominate the landscape of resistome profiling, each offering distinct advantages in terms of resolution and clinical utility.

The gold standard for comprehensive resistome analysis is high-depth shotgun metagenomics sequencing. Unlike 16S rRNA gene sequencing, which is restricted to taxonomic identification, shotgun metagenomics captures the total genomic repertoire of the gut microbiota. This allows for the precise identification of ARGs by mapping sequenced reads against curated reference databases, such as the Comprehensive Antibiotic Resistance Database (CARD) or ResFinder (McArthur et al., 2013). This approach enables researchers to calculate the ARG burden, typically normalized as the number of resistance gene copies per microbial genome or per million reads, providing a high-resolution functional readout of the ecosystem’s resistance potential and its correlation with immunotherapy outcomes.

However, for routine clinical implementation where turnaround time and cost-effectiveness are paramount, high-throughput quantitative polymerase chain reaction (qPCR) arrays represent a more feasible alternative. These arrays utilize pre-validated primer sets to target hundreds of diverse ARGs simultaneously. The primary advantage of qPCR-based profiling lies in its ability to provide absolute quantification of gene copies with a rapid turnaround (24–48 h), making it a practical platform for exploratory profiling and future prospective evaluation prior to ICI therapy, although its predictive value for routine patient stratification has not yet been established.

**FIGURE 1:**
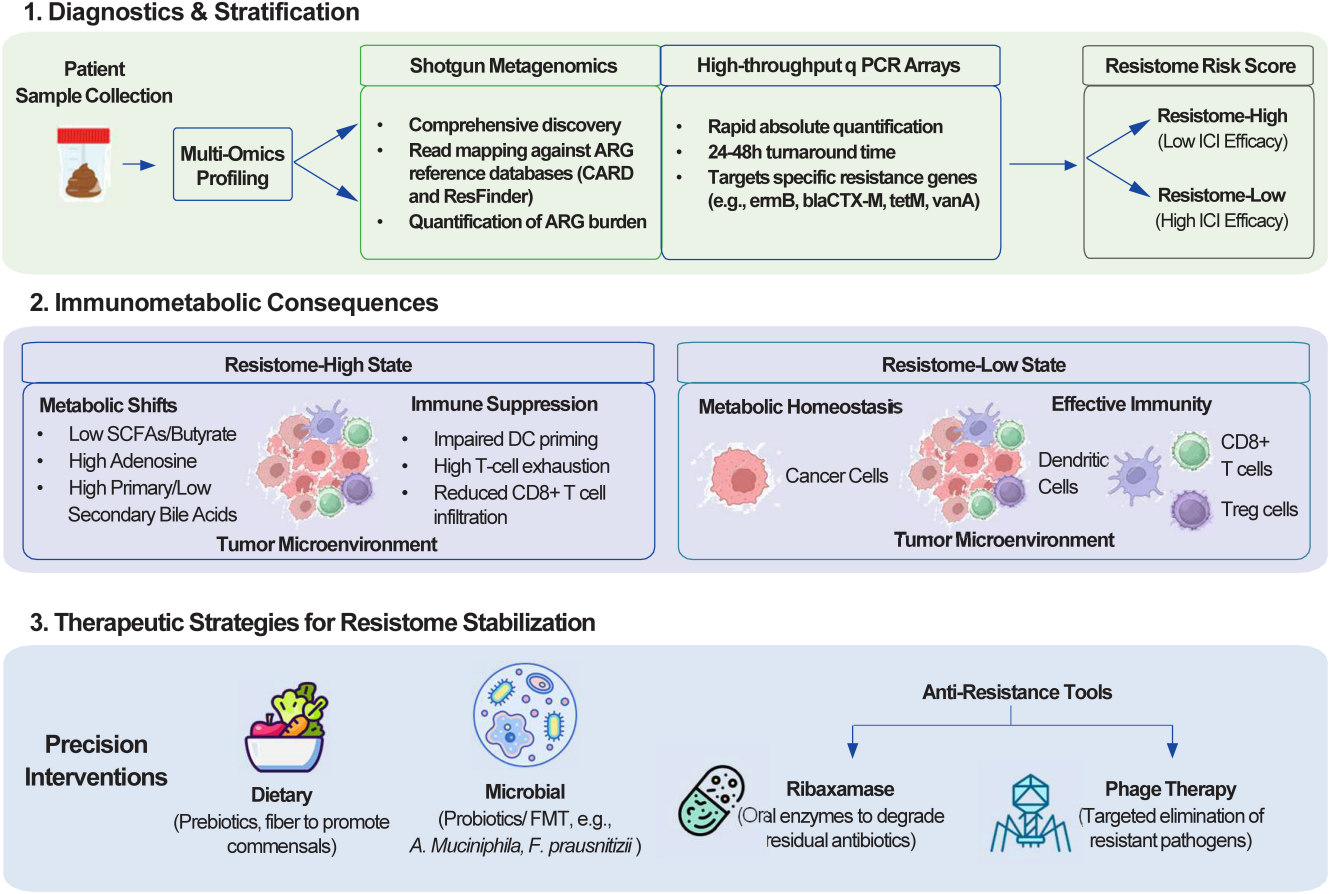
Resistome-centered strategies for preserving immunotherapy effectiveness. Proposed translational framework illustrating how future resistome profiling may support immunotherapy risk stratification following clinical validation. A putative “Resistome-High” state is characterized by immunometabolic alterations associated with impaired anti-tumor immunity, including short-chain fatty acid (SCFA) depletion and elevated adenosine signaling. The conceptual Resistome Risk Score is proposed to identify patients at increased risk before immune checkpoint inhibitor (ICI) therapy. Potential microbiome-targeted interventions, including prebiotics, next-generation probiotics, and anti-resistance approaches such as ribaxamase, may help stabilize the gut ecosystem and preserve treatment efficacy.

Recent advances in sample preservation and logistics have improved the clinical feasibility of resistome-based diagnostics. Nevertheless, no prospectively validated resistome-based score has yet been established for routine clinical use in patients receiving ICIs. Thus, the proposed “Resistome Risk Score” should be regarded as an investigational framework rather than an established clinical assay (Figure 1). A future resistome-based composite score could incorporate several feature domains, including (i) total ARG abundance, (ii) ARG richness or diversity, (iii) class-specific resistance burden, and (iv) the abundance of candidate ARGs that have shown preliminary associations with poorer ICI outcomes, such as lnuC, msrD, ermG, fosA, and aph(6). For shotgun metagenomics, these features may be normalized using read-depth- and gene-length-adjusted metrics, whereas qPCR-based platforms may report absolute or relative gene abundance normalized to total bacterial load, such as 16S rRNA gene copies, standardized DNA input, or stool mass. However, the weighting of individual variables, the optimal cutoffs, and the relative contribution of gene-level versus class-level features remain to be established. Importantly, any threshold would likely require tumor type, treatment, and exposure window-specific calibration rather than a universal cutoff. In addition, pre-analytical and biological sources of variability, including stool collection procedures, storage conditions, sequencing batch effects, diet, and prior antibiotic exposure, would need to be harmonized prospectively or adjusted analytically.

## 3 Part II. Immunometabolic mechanisms and translational strategies

### 3.1 Immunometabolic links between resistome dynamics and anti-tumor immune suppression

Antibiotic exposure can shift the gut microbiome toward a resistome-enriched state, characterized by the depletion of beneficial commensals and the expansion of ARG-harboring pathobionts. Rather than viewing these changes as a purely taxonomic phenomenon, their downstream consequences can be conceptualized within three major immunometabolic axes: (i) SCFA depletion with altered dendritic-cell and T-cell programming, (ii) bile acid remodeling with disrupted the farnesoid X receptor (FXR)/Takeda G-protein-coupled receptor 5 (TGR5) signaling, and (iii) perturbed purine/adenosine metabolism with downstream T-cell suppression (Gopalakrishnan et al., 2018; Iwan et al., 2024). These pathways are biologically interconnected, but separating them analytically improves clarity and helps define how microbiome disruption may impair the immune context required for effective ICI responses.

#### 3.1.1 SCFA depletion and dendritic cell/T-cell programming

Antibiotic-associated microbiome disruption commonly reduces commensal bacteria producing SCFAs, particularly butyrate. In the context of a resistome-enriched gut ecosystem, the loss of these taxa is expected to diminish SCFA availability and alter microbial metabolic output (Kim, 2023). SCFAs are not only a major energy source for colonocytes, but also signal through histone deacetylase-dependent and receptor-mediated pathways that influence dendritic-cell maturation, immune tolerance, and T-cell differentiation (Koh et al., 2016). Reduced butyrate availability may therefore weaken dendritic-cell activation and compromise the quality of CD8 T-cell priming, while also destabilizing regulatory T-cell homeostasis (Furusawa et al., 2013; Smith et al., 2013). From an immunotherapy perspective, this shift is relevant because dendritic cells are central to antigen presentation and cross-priming, and impaired early immune programming may reduce the capacity of checkpoint blockade to reactivate productive antitumor responses (Liu et al., 2023). Thus, the SCFA axis provides one plausible route through which antibiotic-driven microbiome disruption may translate into a less immunologically permissive state for ICI efficacy.

#### 3.1.2 Bile acid remodeling and FXR/TGR5 signaling

**FIGURE 2:**
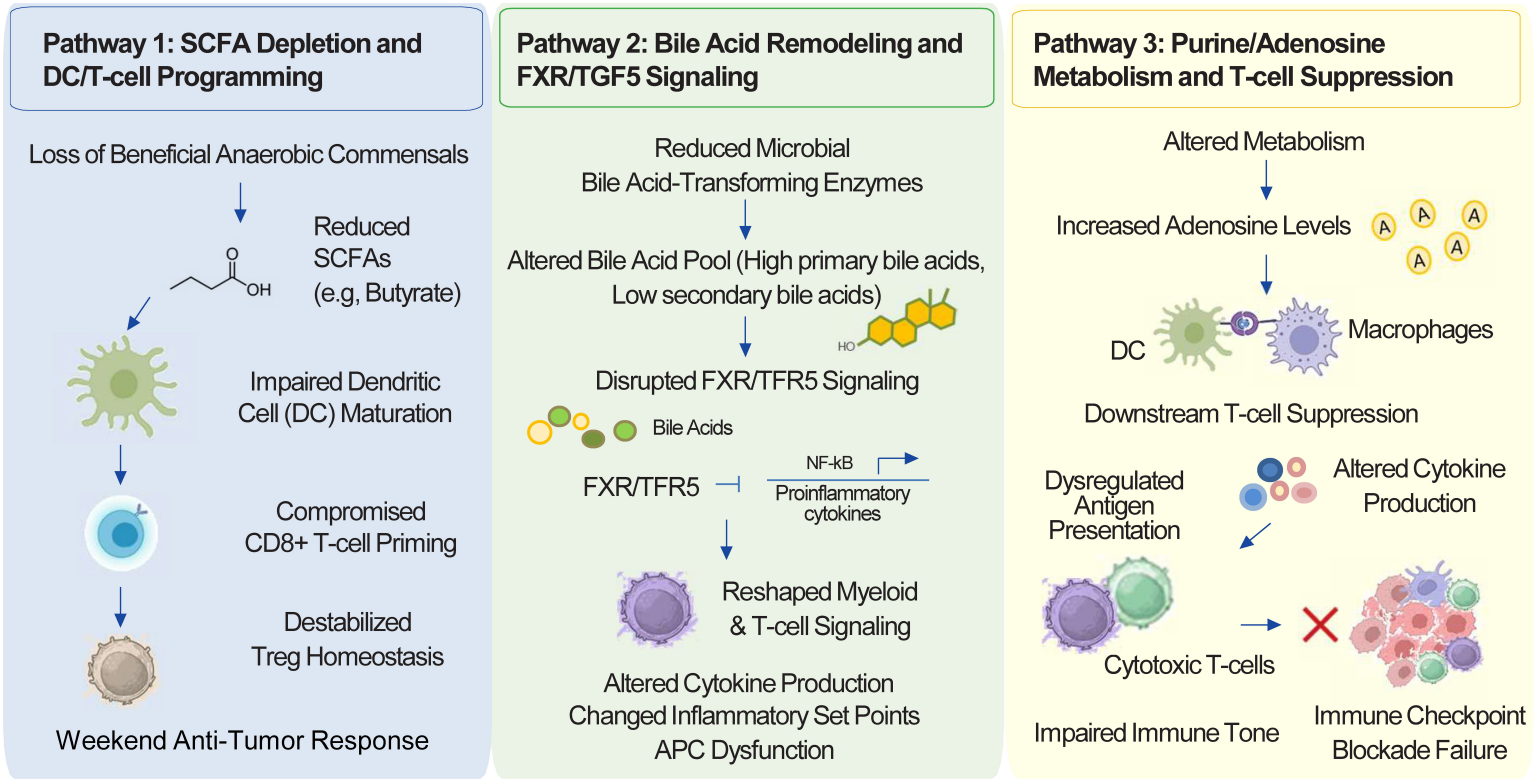
Impact of antibiotic exposure on anti-tumor immunity through gut microbiome. Schematic illustrating how resistome expansion suppresses anti-tumor immunity through three major immunometabolic axes: (i) depletion of commensal-derived short-chain fatty acids (SCFAs), leading to impaired regulatory T-cell stability; (ii) disruption of bile acid signaling, resulting in impaired dendritic-cell antigen presentation through host receptors such as FXR and TGR5; and (iii) enrichment of opportunistic pathogens associated with extracellular adenosine accumulation and T-cell exhaustion. Collectively, these alterations increase the immune activation threshold and contribute to therapeutic resistance.

Antibiotic-driven ecological disruption may also remodel microbial bile acid metabolism by reducing the enzymatic breadth required to convert primary bile acids into secondary bile acids (Godlewska et al., 2022). Consistent with this mechanism, experimental studies have shown that antibiotic-induced microbiome disruption alters the intestinal bile acid pool and increases susceptibility to *Clostridioides difficile* expansion, illustrating how antibiotic-driven ecological shifts reshape bile acid metabolism in the gut (Buffie et al., 2015; Theriot et al., 2014). Because bile acid signaling is mediated through host receptors such as FXR and TGR5, changes in microbial bile acid transformation can influence immune tone beyond the intestine (Ridlon et al., 2016). In a resistome-enriched state, altered bile acid pools may reshape signaling in dendritic cells, macrophages, and T cells, with downstream effects on cytokine production, inflammatory set points, and antigen-presenting cell function (Pathak et al., 2017). These changes are especially relevant in antitumor immunity because dysregulated antigen presentation and myeloid-cell signaling can weaken the immune context required for effective checkpoint blockade. Although the exact bile acid signatures associated with poor ICI response remain to be defined, bile acid remodeling provides a mechanistically distinct axis linking antibiotic-associated microbiome disruption to altered host immune regulation. Antibiotic exposure can enrich microbial populations carrying resistance determinants such as β-lactamase genes (e.g., blaTEM, blaCTX-M), macrolide resistance genes (ermB), and tetracycline resistance genes (tetM), resulting in reshaping the functional composition of the gut microbiome (Kang et al., 2021; Larabi et al., 2023). This shift can alter the production of secondary bile acids and consequently influence dendritic cell activation, cytokine production, and T-cell differentiation, thereby affecting antitumor immune responses (Hang et al., 2019; Jia et al., 2025; Song et al., 2020). Together, these molecular correlations support the hypothesis that the gut resistome may serve as a potentially functional marker to immunotherapy failure across diverse cancer contexts (Figure 2).

#### 3.1.3 Purine/adenosine metabolism and T-cell suppression

**FIGURE 3:**
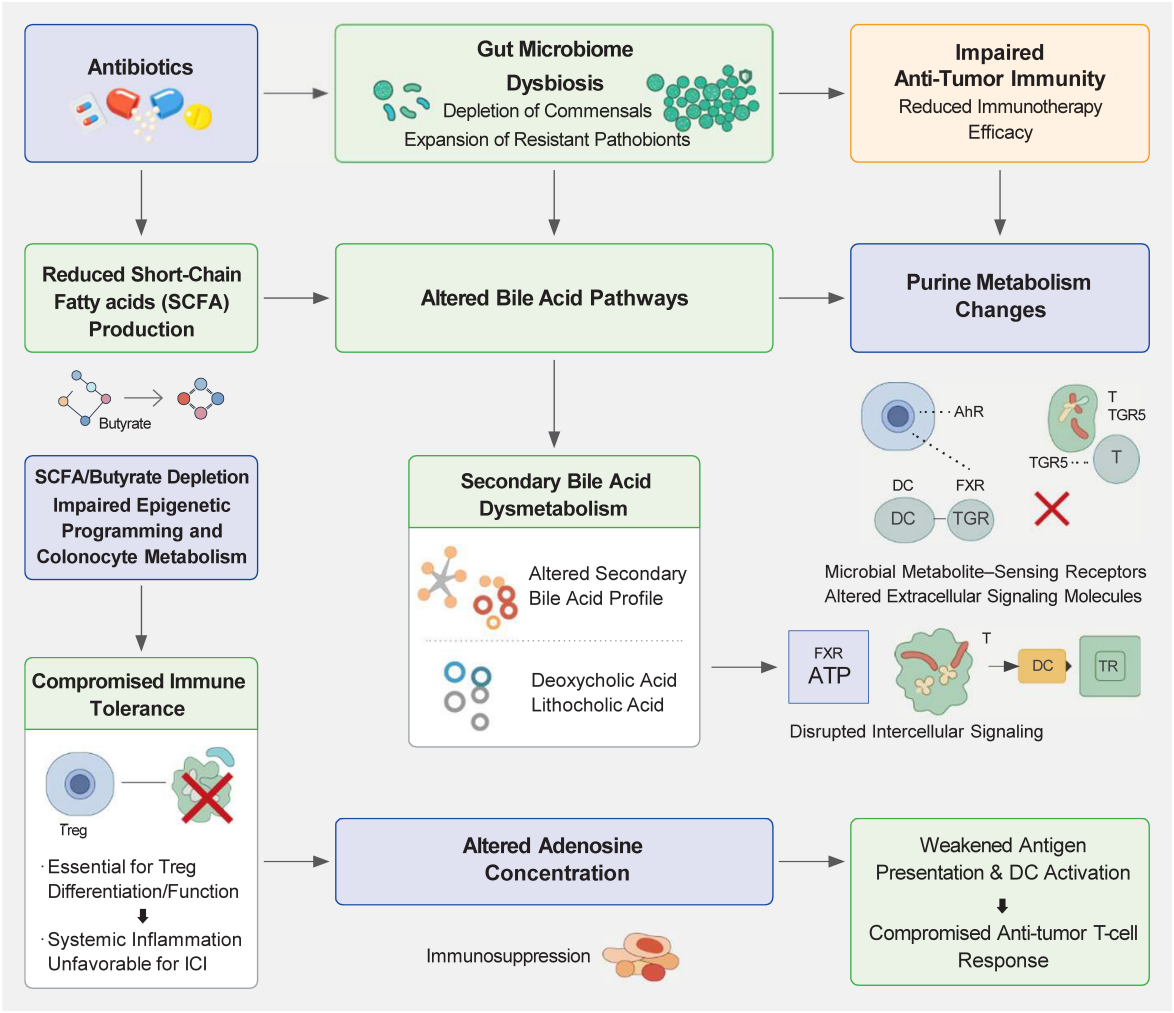
Molecular pathways linking antibiotic exposure, gut dysbiosis, and impaired anti-tumor immunity. Schematic illustrating how antibiotic-induced gut microbiome dysbiosis may disrupt host immunometabolic signaling, impair anti-tumor immune responses, and ultimately reduce immunotherapy efficacy.

Resistome-associated shifts in the gut microbiome involve disturbed microbial purine metabolism and the downstream accumulation of immunosuppressive purine metabolites, particularly adenosine. Antibiotic exposure often favors expansion of opportunistic, ARG-enriched pathobionts, including *Enterococcus*, *Escherichia*, and *Klebsiella* species, many of which have active purine salvage and nucleotide metabolic pathways (Kang et al., 2021; Pamer, 2016; Ubeda et al., 2010). In this setting, microbiome restructuring may alter extracellular purine flux and contribute to an adenosine-rich immune microenvironment (Emery et al., 2025; Gélinas et al., 2021). Adenosine is a well-established suppressor of antitumor immunity. It can inhibit antigen-presenting cell function, raise the activation threshold of T cells, and promote dysfunctional or exhaustion-associated T-cell states (Hesse et al., 2024). Disruption of these metabolic pathways can modify the local concentration of purine metabolites and alter the activation threshold of immune cells (Michaels and Madsen, 2023). Increased extracellular adenosine signaling suppresses dendritic cell activation and impairs antigen presentation, thereby weakening the initiation of cytotoxic T-cell responses (Allard et al., 2016; Boison and Yegutkin, 2019; Ohta and Sitkovsky, 2014). Disruption of microbiome-derived purine metabolism may therefore raise the threshold for immune activation and compromise the early immune priming required for effective checkpoint blockade (Figure 3).

Although these three pathways are biologically interconnected, distinguishing SCFA-dependent immune priming, bile acid–receptor signaling, and purine/adenosine-mediated suppression reduces redundancy and clarifies how microbiome disruption may influence ICI responsiveness through metabolite-linked immune mechanisms. On this basis, the next section discusses translational strategies aimed at stabilizing favorable resistome states and preserving microbiome-dependent immune function.

### 3.2 Strategies to stabilize favorable resistome states

#### 3.2.1 Dietary strategies: prebiotics

Dietary strategies primarily focus on the use of Prebiotics. These involve providing fermentable dietary fibers, such as inulin and oligosaccharides, which are designed to selectively promote the growth of beneficial microbial populations, specifically SCFA-producing microbes (Carlson et al., 2018; Duan et al., 2023; Silva et al., 2020). The introduction of these fibers can accelerate the recovery of beneficial microbial communities after antibiotic exposure. In contrast, antibiotic-induced dysbiosis often promotes expansion of opportunistic taxa that frequently harbor antibiotic resistance genes, including *Enterococcus faecium*, *Escherichia coli*, and *Klebsiella pneumoniae*, which are commonly enriched in disrupted gut ecosystems (Ubeda et al., 2010). By restoring SCFA-producing commensals, prebiotic supplementation may limit the ecological space available for these resistance-associated microbes and help stabilize microbiome structure. The overarching goal of this approach is to stabilize the gut environment and successfully restore immune-regulatory function that was compromised by antibiotic exposure. However, probiotics should not be assumed to uniformly restore microbiome function after antibiotics. Human data indicate that some probiotic formulations may delay endogenous microbiome reconstitution, and observational studies in melanoma suggest that non-specific probiotic use may be associated with lower microbial diversity and less favorable ICI outcomes.

#### 3.2.2 Microbial strategies

Microbial strategies include next-generation probiotics and fecal microbiota transplantation (FMT). Next-generation probiotics involve the targeted administration of bacterial strains with defined metabolic functions, such as SCFA production or bile acid transformation. Examples include *Akkermansia muciniphila* and *Faecalibacterium prausnitzii*, which have been associated with restoration of beneficial microbial metabolism and improved antitumor immune responses (Matson et al., 2018; Mohiuddin et al., 2021; Sivan et al., 2015; Vétizou et al., 2015). FMT represents another strategy aimed at restoring microbial ecosystem structure and metabolic capacity. By transferring an intact microbial community from a healthy donor, FMT has been investigated as a potential approach to reverse antibiotic-associated dysbiosis and improve responsiveness to immune checkpoint inhibitors (Gopalakrishnan et al., 2018; Novelle et al., 2025). In a proof-of-concept clinical study, FMT from immunotherapy responders reshaped the gut microbiome and induced clinical responses in a subset of melanoma patients who were previously resistant to anti-PD-1 therapy (Baruch et al., 2021). Similarly, a parallel study demonstrated that FMT combined with anti-PD-1 therapy restored microbial diversity and reprogrammed tumor immune responses, leading to renewed sensitivity to checkpoint blockade in treatment-refractory patients (Davar et al., 2021). These findings suggest that restoring microbial community structure through FMT may also help rebalance the gut resistome and recover immunometabolic functions that support effective antitumor immunity. In this context, rationally designed microbial consortia composed of defined commensal strains, together with engineered bacteria designed to modulate immune responses, may represent controlled strategies to stabilize microbiome function and enhance immune checkpoint inhibitor efficacy (Tanoue et al., 2019). Importantly, microbiome-directed interventions are not equally mature. FMT currently has the strongest early clinical signal in the immunotherapy setting, particularly in ICI-refractory melanoma, whereas conventional probiotics, next-generation live biotherapeutics, and engineered microbial approaches remain considerably less validated in oncology. Even when biologically plausible, these platforms should be interpreted cautiously in immunocompromised patients because live microbial products may pose risks of bacteremia, fungemia, excessive immune activation, or unpredictable colonization.

#### 3.2.3 Anti-resistance strategies

Another approach focuses on limiting the expansion of the gut resistome during necessary antibiotic treatment. Because antibiotic exposure can rapidly select for resistant microbial populations and promote horizontal transfer of antibiotic resistance genes, strategies that reduce these processes may help preserve microbiome stability and immune function (Maharramov et al., 2025).

One potential strategy involves microbiome-protective enzymes such as ribaxamase, an orally administered β-lactamase designed to degrade residual β-lactam antibiotics in the intestine and thereby prevent antibiotic-induced microbiome disruption and the emergence of resistant organisms (Narendrakumar et al., 2022). Ribaxamase co-administration with intravenous ceftriaxone has been shown to protect the gut microbiome and reduce the incidence of *Clostridioides difficile* infection in clinical studies, demonstrating the feasibility of protecting the intestinal microbiome during antibiotic therapy (Kokai-Kun et al., 2017, 2019). However, ribaxamase and other microbiome-protective co-therapies should be viewed as proof-of-principle rather than oncology-ready interventions. Ribaxamase has evidence for microbiome protection during intravenous β-lactam exposure, but its effect on cancer immunotherapy outcomes has not been established. Phage therapy and antibiotic potentiators are even earlier in development, with limited oncology-specific clinical data.

Bacteriophage therapy is another strategy that selectively targets antibiotic-resistant bacteria while sparing beneficial commensal microbes (Kortright et al., 2019). Because bacteriophages exhibit high host specificity, phage therapy has been proposed as a precision antimicrobial strategy capable of eliminating resistant pathogens without broadly disrupting microbiome composition (Dedrick et al., 2019; Kortright et al., 2019). By limiting the expansion of resistance gene-carrying microbes, such approaches may help maintain microbial metabolic functions that support antitumor immunity. In addition, antibiotic potentiators and resistance inhibitors have been developed as adjuvant therapies that block resistance acquisition pathways or reduce the fitness of resistant bacteria while preserving the therapeutic effects of antibiotics (Joseph et al., 2025; Klobucar and Brown, 2022; Narendrakumar et al., 2022).

Together, these precision antimicrobial strategies support a resistome-centered framework for intervention. Such strategies may enable patient stratification and therapeutic development aimed at maintaining immunotherapy efficacy in patients who require antibiotic treatment. This comprehensive schematic illustrates the integrated clinical workflow for stabilizing the gut resistome to preserve immunotherapy efficacy. Standardized diagnostic pathways utilizing high-depth metagenomics or rapid high-throughput qPCR are employed to calculate a dynamic resistome risk score, which serves as a biomarker for patient stratification prior to ICI initiation. Expansion of ARGs triggers distinct immunometabolic consequences. A resistome-high state drives suppression via depleted SCFAs, altered primary-to-secondary bile acid balance, and inefficient T-cell priming, leading to exhaustion. Targeted therapeutic strategies categorized as dietary, microbial, and anti-resistance tools aim to stabilize the resistome, restore metabolic homeostasis, and recover durable anti-tumor immunity in patients requiring systemic antibiotics (Figure 1).

## 4 Conclusions and perspectives

**FIGURE 4:**
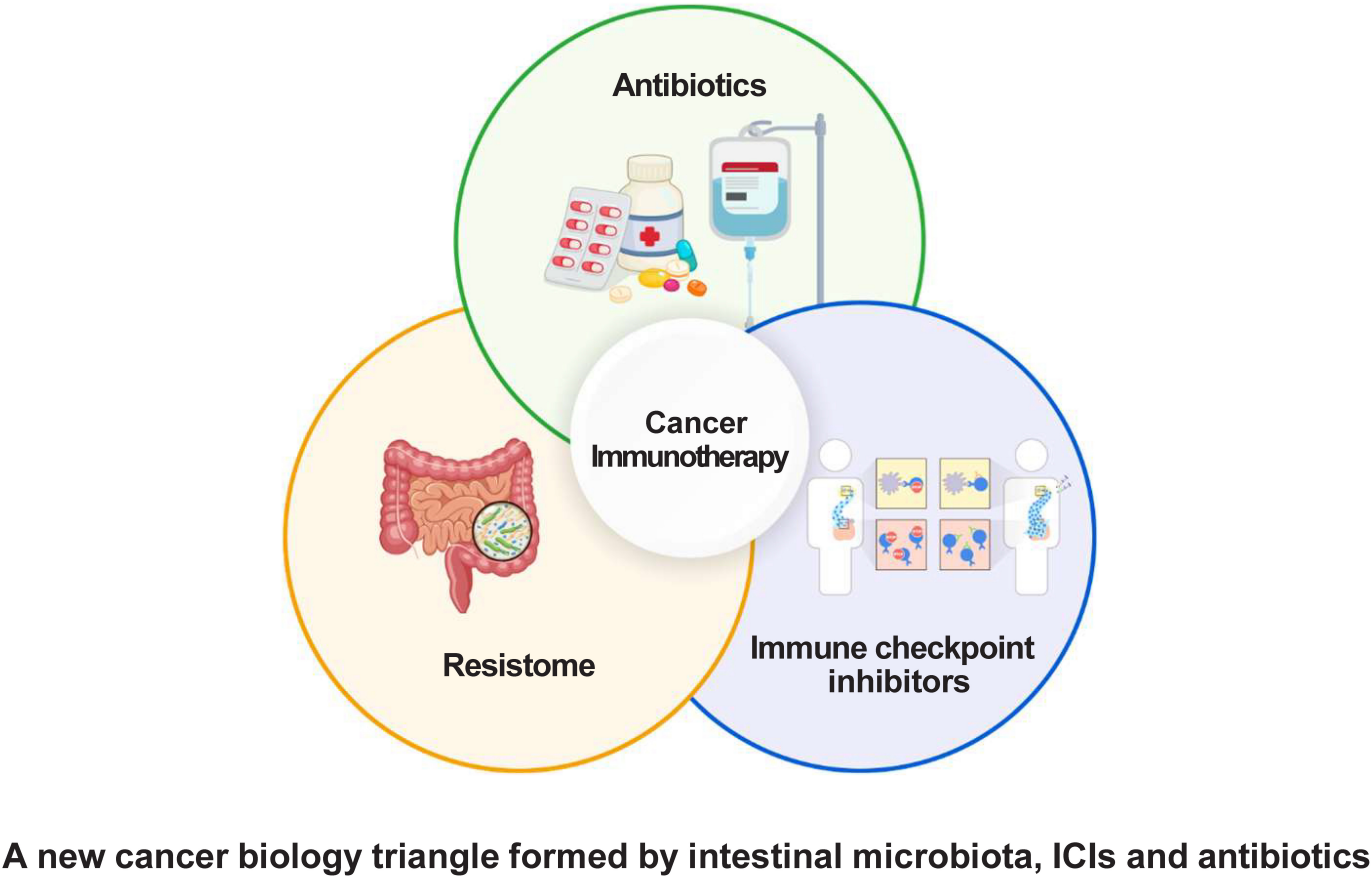
A new cancer biology triangle formed by intestinal microbiota, ICIs and antibiotics. Schematic illustrating the interconnected relationship among antibiotics, the intestinal microbiota, and immune checkpoint inhibitors (ICIs) in cancer immunotherapy. This conceptual framework highlights how antibiotic-mediated alterations in the gut microbiota may significantly influence the therapeutic efficacy of ICIs.

Accumulating clinical evidence consistently demonstrates that systemic antibiotic exposure, particularly within the 2 months preceding ICI therapy, is consistently associated with reduced treatment efficacy across multiple cancer types. This adverse effect cannot be fully explained by changes in microbial taxonomy alone. Rather, antibiotic-driven expansion of the gut resistome, defined as the collective pool of ARGs, provides a useful functional framework linking antibiotic use to impaired antitumor immunity (Figure 4). Antibiotic exposure frequently promotes the expansion of opportunistic bacterial taxa that harbor diverse resistance determinants, including members of the genera *Enterococcus*, *Escherichia*, and *Klebsiella*, which commonly dominate disrupted gut ecosystems following antibiotic treatment. Such microbiome restructuring has been associated with reduced clinical responses and shorter survival in patients receiving immune checkpoint inhibitors (Gopalakrishnan et al., 2018; Pinato et al., 2019). Resistome expansion reshapes key immunometabolic pathways by depleting SCFA-producing bacteria, promoting the expansion of opportunistic pathogens such as *Clostridioides difficile*, disrupting bile acid signaling through immune-regulatory receptors, and altering microbial-driven purine metabolism, collectively impairing antigen presentation, promoting T-cell exhaustion, and suppressing cytotoxic immune programs.

These findings highlight the gut resistome as a promising and previously underexplored candidate determinant of immunotherapy outcomes and help explain why the timing of antibiotic exposure is central to clinical risk stratification. Importantly, advances in metagenomic sequencing and ARG-targeted profiling platforms have made resistome analysis increasingly feasible in translational and clinical research. ARG abundance and diversity can now be quantified from stool-derived microbial DNA using shotgun metagenomics or high-throughput qPCR approaches, with sequence alignment against curated databases such as the CARD and ResFinder (Lanza et al., 2018; McArthur et al., 2013; Zankari et al., 2012). Compared with conventional taxonomic profiling, resistome analysis may provide a more functionally informative readout of microbiome disruption by capturing the selective pressure imposed by antibiotic exposure and the expansion of resistance-associated pathobionts. Although challenges related to standardization, cost, and interpretation remain, ongoing improvements in sequencing technologies and bioinformatic pipelines are expected to facilitate broader clinical implementation. Moving forward, the field must shift beyond taxonomic biomarkers toward incorporating resistome profiling into prospective clinical trials to improve patient selection and therapeutic decision-making. Stabilization of the gut resistome may represent a future translational objective, with potential interventions including dietary modulation, microbiome-based therapies, and anti-resistance strategies to mitigate ARG expansion during unavoidable antibiotic use. Accordingly, future translational work should distinguish among low-risk supportive approaches (for example, diet-based modulation), early clinical ecosystem-level strategies (such as FMT), and emerging precision platforms (including live biotherapeutics, phages, and antibiotic adjuvants). These approaches differ substantially in evidentiary maturity, safety profile, regulatory complexity, and implementation feasibility. Establishing a unified, resistome-centered framework will be essential for advancing precision immuno-oncology and preserving immunotherapy efficacy in patients who require antibiotics.

## 5 Limitations of study

Despite accumulating clinical and experimental evidence linking antibiotic-induced microbiome disruption to reduced immunotherapy efficacy, several important limitations remain. First, the clinical evidence associating antibiotic exposure with inferior ICI outcomes is heterogeneous and potentially confounded, because many retrospective studies do not uniformly account for antibiotic indication, infection severity, hospitalization, concomitant steroid use, performance status, tumor burden, or line of therapy. Second, exposure definitions vary substantially across studies, particularly with respect to whether antibiotics were administered before, during, or after ICI initiation, and key treatment characteristics such as duration, dose, route, antibiotic class, and the use of ICI monotherapy versus chemoimmunotherapy are often incompletely reported, thereby complicating cross-study comparisons and limiting causal inference. Third, current evidence supporting the role of the gut resistome in cancer immunotherapy is largely associative, and direct molecular mechanisms connecting ARG expansion to impaired antitumor immunity have not yet been fully established. Fourth, microbiome composition and resistome dynamics exhibit substantial inter-individual variability shaped by host immunity, tumor stage, environmental exposure, diet, and prior treatment history. Moreover, the relative contribution of the gut resistome may differ between pre-metastatic and advanced metastatic settings, where immune landscapes and therapeutic susceptibilities are fundamentally distinct. In addition, because host immune responses can themselves influence microbial reconstitution following antibiotic exposure, bidirectional interactions between immunity and the microbiota should be carefully considered. Finally, from a translational perspective, no prospectively validated resistome-based composite score is currently available for routine oncology practice, and any future scoring framework will require harmonized pre-analytical workflows, including standardized stool collection, preservation, DNA extraction, and platform-specific normalization, together with rigorous external validation across independent cohorts and likely tumor-specific calibration before clinical implementation can be considered. Accordingly, future studies integrating longitudinal microbiome and resistome profiling, mechanistic immune analyses, metabolomics, and standardized analytic pipelines will be necessary to establish causal relationships, refine risk stratification, and determine clinical utility.
